# A rapid and efficient method for enriching mitochondrial DNA from plants

**DOI:** 10.1080/23802359.2018.1438856

**Published:** 2018-02-15

**Authors:** Mackenzie M. Strehle, Emma Purfeerst, Alan C. Christensen

**Affiliations:** School of Biological Sciences, University of Nebraska, Lincoln, NE, USA

**Keywords:** DNA extraction, DNA purification, qPCR, Arabidopsis

## Abstract

Current mitochondrial purification techniques are tedious and protracted due to their emphasis on recovering physiologically active mitochondria. However, for studies that are exclusively interested in isolating mitochondrial DNA (mtDNA) for applications such as PCR and sequencing, respiring mitochondria − and the complex procedures that stem from the need to retain their function − are unnecessary. Still, global DNA extraction methods have proven insufficient for mitochondrial DNA isolation because nuclear mitochondrial DNA segments (NUMTs) pose unique challenges to accurate mtDNA quantification and characterization. We present a rapid and simple extraction technique that maximizes recovery of mitochondrial DNA from plant cells, while minimizing the presence of nuclear DNA. Through real-time PCR, we show that this method provides a significant increase in the enrichment of mitochondrial DNA compared to that of nuclear DNA in both *Arabidopsis thaliana* and *Brassica rapa*. This method has important implications for future mitochondrial DNA analyses as it possesses few procedural limitations and minimizes the analytical problems typically associated with mtDNA purification by other techniques.

## Introduction

For applications involving PCR and sequencing, the presence of the entire mitochondrial genome or fragments of it (Lin et al. 1999; Stupar et al. 2001; Richly and Leister 2004; Hazkani-Covo et al. 2010) in the nuclear chromosomes can confound results that depend on strict amplification of the mitochondrial copies of DNA. Further, without purification or enrichment of mtDNA, nuclear DNA will comprise the majority of NextGen DNA sequencing reads, thus increasing the total amount of sequence data required and the overall cost to sequence the mitochondrial (or plastid) genome. Although there are simple and efficient procedures for extracting total plant DNA for use in polymerase chain reactions (PCR) (Edwards et al. 1991) and NextGen sequencing, these methods do not always work well for specifically extracting mitochondrial DNA (mtDNA). Treating purified mitochondria with DNAses in order to degrade contaminating nuclear DNA is also problematic because this often results in simultaneous degradation of the mitochondrial DNA (Li et al. 2006).

Due to their emphasis on retaining physiologically active mitochondria for use in metabolic or respiration experiments, existing procedures (Hayes et al. 1991) may not provide good yields of mtDNA suitable for molecular biology applications and they may retain nuclear DNA. Additionally, these protocols tend to be lengthy and tedious compared with standard DNA purification procedures.

We have developed a rapid and efficient method for significant enrichment of mitochondrial DNA relative to nuclear DNA in plants, and have tested it on two species: *Arabidopsis thaliana* and *Brassica rapa*. The procedure also significantly enriches for plastid DNA.

## Materials and methods

### Plants

*Arabidopsis thaliana* ecotype Columbia-0 was obtained from the Lehle Seed Company (Round Rock, TX). *Brassica rapa* was obtained from the USDA-ARS North Central Regional Plant Introduction Station, Ames, Iowa 50011-1170 (accession PI431573).

### Total DNA purification

Preparation of total DNA from both species was performed using the Qiagen DNeasy Plant DNA Mini Kit. DNA prepared in this manner was compared to the mitochondrial enrichment protocol described below.

### Mitochondrial DNA purification

1. Leaves from *Arabidopsis thaliana* or *Brassica rapa* were removed and frozen at −80 °C for 1 h.
2. The frozen leaves were ground in a chilled mortar and pestle with cold (4 °C) grinding medium (350 mM mannitol, 30 mM MOPS, 1 mM EDTA, 50 μM PVPP, 11.2 μM L-cysteine; pH 7.6) at a volume of 2 mL medium per gram of leaf tissue.
3. The pulp was filtered through cheesecloth into a small beaker. The mortar was rinsed with cold grinding medium to recover residual leaf matter and the additional pulp was filtered through the cheesecloth.
4. The crude product was filtered through a 0.45 μm syringe filter into clean microcentrifuge tubes and frozen at −80 °C for one hour.
5. After thawing the filtrate at room temperature, it was inverted two to three times and centrifuged in the cold at 5050g for two minutes.
6. The supernatant was transferred to new microcentrifuge tubes and centrifuged in the cold at 20,000*g* for 5 min.
7. The resulting supernatant was discarded and the pellets were resuspended in 500 μL wash medium (300 mM mannitol, 20 mM MOPS, 1 mM EDTA; pH 7.2). This suspension was centrifuged at 20,000g for one minute, and the supernatant was discarded.
8. The pellets were resuspended in 30 μL TE (10 mM Tris, 1 mM EDTA, pH 8) and vortexed briefly to break up remaining fragments.
9. DNA from the resuspended pellets was purified using the QIAquick PCR Purification Kit (Qiagen, Valencia, CA) following the instructions from the manufacturer and stored at −20 °C.

### Quantitative PCR analysis

Real-time PCR was performed on 4 biological replicates of DNA extracted by the method outlined above and 4 biological replicates of DNA extracted by the Qiagen DNeasy Plant Mini Kit (Qiagen, Valencia, CA). Each replicate was amplified with three pairs of primers that were designed to flank sequences in the nuclear, chloroplast, and mitochondrial genomes, respectively. All three primer pairs were run with the same parameters, which included an initial denaturation step at 95 °C for 10 min, followed by a three-step amplification cycle consisting of denaturation at 95 °C for 10 s, annealing at 60 °C for 15 s, and extension at 72 °C for 20 s. This three-step cycle was repeated for 45 cycles. Primer sequences and amplicon size are listed in Table 1. Primers were purchased from ThermoFisher (Waltham, MA). Reactions were performed in triplicate in 96-well plates using a Bio-Rad CFX96 thermocycler (Bio-Rad, Hercules, CA) after diluting each sample to a concentration of 2ng/μL. EvaGreen 2× qPCR mastermix (obtained from MidSci) was used for all reactions with a total reaction volume of 12 μL/well (Wang et al. 2006; Navarro et al. 2015). Cq values were generated by the Bio-Rad CFX96 using the regression setting (Supplemental data). The Cq values for the three technical replicates of each biological replicate were averaged and analyzed as described below.

**Table 1.** Primer pairs used for real-time quantitative PCR of nuclear, mitochondrial and plastid DNA, along with annealing temperature and amplicon size for each pair.

Compartment	Gene, accession	Primers (5′–3′)	*T*_m_ (°C)	Amplicon size
Nucleus	*Leafy*, AF466801.1	CAACGAAGGTGAGGATGACG GATAAACGGATGCTCCCTCTG	60	88 bp
Plastid	*matK*, AP000423.1	TCACGGAAGATGCATTCTGG TGACCTTTTGCGATTGAAACC	60	119 bp
Mitochondrion	*nad9*, JF729201.1	AGTAAGTCTATTTCCATCAGCCG CACTCAGAGGAAGGTCTTTTCG	60	149 bp

### Statistical analysis

Fold change in mitochondrial DNA content relative to nuclear DNA content was calculated for each *A. thaliana* biological replicate (average of three technical replicates) using the equation 2^−ΔCq^ where ΔCq is equal to the average Cq value for mitochondrial DNA amplification (Cq_mito_) minus the average Cq value for nuclear DNA amplification (Cq_nuc_) (Livak and Schmittgen 2001). An unpaired, two-tailed *T*-test was performed using the fold change values for each set of replicates to compare the relative amount of mitochondrial DNA recovered by the Qiagen DNeasy Mini Kit compared with the method outlined in this paper.

The same calculations were applied to the analysis of mitochondrial DNA recovery in *B. rapa* purified by each procedure; however, 10 out of the 12 technical replicates failed to amplify with the nuclear primers in the DNA samples extracted by our method, and a valid Cq value could not be determined. Because the qPCR runs were done with 45 cycles, we used 45 as a minimal estimate of the Cq value for these 10 replicates. This estimate gives the minimum possible difference with the mitochondrial Cq values and a conservative estimate of the fold-change. Although valid statistical comparisons are not possible with the undetectable nuclear DNA, the standard deviations of the replicates of the mitochondrial and plastid DNA samples are reported. These calculations were also performed with the Cq values of the plastid primers for both *A. thaliana* and *B. rapa* purified by each procedure.

## Results

### Relative copy number of DNA from nuclei, mitochondria and plastids

#### Arabidopsis thaliana

Using the Qiagen DNeasy kit (Qiagen, Valencia, CA), we obtained an average DNA yield of 16 μg (±2.7) from the four biological replicates starting with 0.1 g of fresh leaves. The method described in this paper resulted in an average yield of 515 ng (±19) starting from approximately 1g of fresh leaves. The 260/280 ratio for each biological replicate exceeded 1.8. The qPCR data are shown in Supplemental Data. Table 2 shows the average and standard deviation of the Cq values from the biological replicates using the nuclear, mitochondrial and plastid DNA primers.

**Table 2.** Results of qPCR analysis. Averages and standard deviations of Cq values of the biological replicates for the two species and two methods used are shown for the primer pairs from each cellular compartment.

	Cq values, avg ± SD			Copy number relative to nucleus	
Species and method	Mitochondria	Chloroplast	Nucleus	Mitochondria	Chloroplast
**A. thaliana** DNeasy	21.90 ± 0.26	17.39 ± 0.29	26.27 ± 0.36	20.9 ± 3.06	477 ± 58
*A. thaliana* MitoPrep	23.85 ± 0.37	21.96 ± 0.49	33.00 ± 0.16	604 ± 214*	2171 ± 514*
*B. rapa* DNeasy	19.26 ± 0.12	27.07 ± 0.94	34.31 ± 0.96	42391 ± 32294	314 ± 359
*B. rapa* MitoPrep	23.96 ± 0.19	29.88 ± 0.24	N/A	1350963 (est.)*	25126 (est.)

* *p* < .05.

As can be seen from Table 2, our procedure changed the copy number of mitochondrial DNA from 20.5 copies per copy of the nuclear genome to 604 copies. This is a 30-fold enrichment of the mitochondrial genome. The chloroplast was similarly enriched by six-fold. The enrichment of the mitochondrial DNA from *A. thaliana* was significant with *p* < .05. The chloroplast enrichment was also significant with *p* < .05.

#### Brassica rapa

Using the Qiagen DNeasy kit (Qiagen, Valencia, CA), we obtained an average yield of 1115 ng (±56) of DNA from the four biological replicates starting with 0.1 g of fresh leaves. The method described in this paper resulted in an average yield of 555 ng (±70) starting from approximately 1 g of fresh leaves. The 260/280 ratio for each biological replicate exceeded 1.8. The qPCR data are shown in Supplemental data. Table 2 shows the average and standard deviation of the Cq values from the biological replicates using the nuclear, mitochondrial, and plastid DNA primers.

The results were similar to those obtained with *Arabidopsis thaliana*; however, the fold change resulting from our procedure had to be estimated because the nuclear DNA was frequently undetectable. Of all of the biological and technical replicates, nuclear DNA was only detectable in one of the technical replicates of each of two different biological replicates, and was undetectable in the other 10 samples. We repeated the qPCR with a four-fold higher input of the same samples of DNA, but nuclear DNA remained undetectable in the same 10 samples (data not shown). The relative copy number of mitochondria in *Brassica rapa* was much higher using the DNeasy kit than it was for *A. thaliana*. This may be related to the greater thickness and ‘tougher’ structure of the mature leaves we used from *B. rapa*, which led to greater differential recovery of mitochondria from the grinding process. Nevertheless, our procedure essentially eliminated nuclear DNA from the final product, while retaining a satisfactory yield of DNA (see Table 2).

## Discussion

Plant mitochondrial purification procedures have been developed, but primarily for physiological studies involving intact, respiring mitochondria. For genomics work, only the nucleoid needs to remain intact inside the inner membrane. We modified and simplified a mitochondrial purification procedure (Hayes et al. 1991) to emphasize steps that concentrated the mitochondrial DNA-containing fraction and excluded as much nuclear DNA as possible. Quantitative PCR analysis of our method, compared to total DNA purification, shows a 30-fold enrichment of mitochondrial DNA from *A. thaliana*. In *B. rapa*, although the enrichment is much higher, we can only estimate the fold-change because the nuclear DNA is no longer detectable – which is the ultimate goal of such a method. This procedure does not involve gradient centrifugation, and is fast and easy. The yields are sufficient for PCR applications, as well as for construction of Next-Gen sequencing libraries. The much lower relative copy number of the nuclear DNA means that fewer sequencing reads will be needed, and contamination of the sequence reads by nuclear fragments of mitochondrial or plastid DNA will be at a much lower relative read-depth and will, therefore, not impact assembly or detection of mutations. This procedure is inexpensive, rapid, does not require an ultracentrifuge, and will prove very useful in PCR or sequencing applications of mitochondria or plastids from plants, as you wish.

## Supplementary Material

Alan_Christensen_et_al_supplemental_content.zip
