# Supplementary material for: A rapid and efficient method for enriching mitochondrial DNA from plants
Source: Mitochondrial DNA B Resour. 2018 Feb 15;3(1):239–42. doi: 10.1080/23802359.2018.1438856 (PMC7800896; doi:10.1080/23802359.2018.1438856)
Supplement: Alan_Christensen_et_al_supplemental_content.zip [file TMDN_A_1438856_SM4547.zip › Alan Christensen et al supplemental content.pdf]

Supplemental Table

The worksheet labeled “Raw Data” reports the Cq values obtained from the qPCR experiments, as generated by the Bio-Rad CFX96 using the regression setting, following reactions done as described in Materials and Methods.

The worksheet labeled “Analysis Data” shows the calculations that were done using the raw data to determine the relative copy numbers. Formulas in the cells indicate the exact data used and the calculations, as well as the statistical analysis used. The calculations are described more fully in Materials and Methods.

Raw Data

A.thaliana DNeasy		Cq Values		
	Technical Replicate	Mito	Chloro	Nuc
Biological Replicate 1	A	21.94	17.56	25.91
	B	22.33	17.27	26.21
	C	21.79	17.61	26.04
Biological Replicate 2	A	21.42	17.04	25.75
	B	21.43	16.86	25.8
	C	21.5	16.77	25.83
Biological Replicate 3	A	22.19	17.42	26.56
	B	22.3	17.69	26.75
	C	21.84	17.59	26.43
Biological Replicate 4	A	22.09	17.62	26.5
	B	21.89	17.81	26.73
	C	22.09	17.38	26.73

A.thaliana MitoPrep		Cq Values		
	Technical Replicate	Mito	Chloro	Nuc
Biological Replicate 1	A	22.8	21.73	33.34
	B	23.27	22.08	32.55
	C	23.63	24.28	33.48
Biological Replicate 2	A	24.09	21.69	32.63
	B	24.05	21.63	32.12
	C	24.15	21.47	34.23
Biological Replicate 3	A	24.13	21.57	32.44
	B	24.25	21.27	32.75
	C	24.08	21.46	33.05
Biological Replicate 4	A	24.03	20.58	33.06
	B	23.61	20.95	33.86
	C	24.06	24.85	32.5

B.rapa DNeasy		Cq Values		
	Technical Replicate	Mito	Chloro	Nuc
Biological Replicate 1	A	19.37	26.11	33.28
	B	19.34	26.49	34.58
	C	19.32	25.97	34.4
Biological Replicate 2	A	19.31	26.27	37.33
	B	19.48	25.86	33.75
	C	19.25	26.12	36.71
Biological Replicate 3	A	19.08	28.17	34.75
	B	19.04	27.97	32.78
	C	19.03	27.28	32.88
Biological Replicate 4	A	19.42	28.33	33.7
	B	19.29	28.32	33.74
	C	19.23	27.92	33.78

B.rapa MitoPrep		Cq Values		
	Technical Replicate	Mito	Chloro	Nuc
Biological Replicate 1	A	24.72	28.94	N/A
	B	23.63	29.45	N/A
	C	23.63	30.13	N/A
Biological Replicate 2	A	23.9	29.72	N/A
	B	23.88	30.09	N/A
	C	23.86	29.72	42.43
Biological Replicate 3	A	24.22	30.06	N/A
	B	24.11	30.35	N/A
	C	24.43	29.86	N/A
Biological Replicate 4	A	23.67	29.09	35.45
	B	23.77	30.57	N/A
	C	23.74	30.57	N/A

Analysis Data

A. thaliana

DNeasy	Mito	Chloro	Nuc	Mito-Nuc	Mito Fold Change	Chloro-Nuc	Chloro Fold Change
Biological Replicate 1	22.02	17.48	26.05	-4.03	16.37	-8.57	380.92
Biological Replicate 2	21.45	16.89	25.79	-4.34	20.30	-8.90	478.82
Biological Replicate 3	22.11	17.57	26.58	-4.47	22.16	-9.01	516.75
Biological Replicate 4	22.02	17.60	26.65	-4.63	24.76	-9.05	530.06
Average	21.90	17.39	26.27	-4.37	20.90	-8.89	476.64
Standard Deviation	0.26	0.29	0.36	0.22	3.06	0.19	58.37
**MitoPrep**	**Mito**	**Chloro**	**Nuc**	**Mito-Nuc**	**Mito Fold Change**	**Chloro-Nuc**	**Chloro Fold Change**
Biological Replicate 1	23.23	22.70	33.12	-9.89	948.83	-10.43	1376.38
Biological Replicate 2	24.10	21.60	32.99	-8.90	476.61	-11.40	2696.12
Biological Replicate 3	24.15	21.43	32.75	-8.59	386.23	-11.31	2544.79
Biological Replicate 4	23.90	22.13	33.14	-9.24	604.67	-11.01	2067.02
Average	23.85	21.96	33.00	-9.16	604.08	-11.04	2171.08
Standard Deviation	0.37	0.49	0.16	0.48	213.63	0.38	514.22

T-Test Dneasy vs Mito Prep (*A. thaliana*)

Mito Fold Change	0.0179
Chloro Fold Change	0.0101

B. rapa

DNeasy	Mito	Chloro	Nuc	Mito-Nuc	Mito Fold Change	Chloro-Nuc	Chloro Fold Change
Biological Replicate 1	19.34	26.19	34.09	-14.74	27427.46	-7.90	238.31
Biological Replicate 2	19.35	26.08	35.93	-16.58	98193.05	-9.85	920.75
Biological Replicate 3	19.05	27.81	33.47	-14.42	21920.61	-5.66	50.68
Biological Replicate 4	19.31	28.19	33.74	-14.43	22022.13	-5.55	46.85
Average	19.26	27.07	34.31	-15.04	42390.81	-7.24	314.15
Standard Deviation	0.12	0.94	0.96	0.90	32294.37	1.77	358.67
**MitoPrep**	**Mito**	**Chloro**	**Nuc**	**Mito-Nuc**	**Mito Fold Change**	**Chloro-Nuc**	**Chloro Fold Change**
Biological Replicate 1	23.99	29.51	45.00	-21.01	2106865.33	-15.49	46127.30
Biological Replicate 2	23.88	29.84	44.14	-20.26	1258551.95	-14.30	20171.07
Biological Replicate 3	24.25	30.09	45.00	-20.75	1759417.78	-14.91	30786.28
Biological Replicate 4	23.73	30.08	41.82	-18.09	279018.26	-11.74	3420.52
Average	23.96	29.88	43.99	-20.03	1350963.33	-14.11	25126.29
Standard Deviation	0.19	0.24					

T-Test Dneasy vs Mito Prep (*B. rapa*)

Mito Fold Change	0.0458
Chloro Fold Change	0.0700
